# Characteristics of Pediatric Dental Treatment Provided under General Anesthesia in Dubai, United Arab Emirates: A Retrospective Analysis

**DOI:** 10.1155/2022/9900775

**Published:** 2022-09-22

**Authors:** Mohammad Abdo, Manal Al Halabi, Iyad Hussein, Anas Salami, Amar H. Khamis, Mawlood Kowash

**Affiliations:** Mohammed Bin Rashid University of Medicine and Health Sciences, Hamdan Bin Mohammed College of Dental Medicine, Dubai Healthcare City, Dubai, UAE

## Abstract

**Background:**

Dental general anesthesia (DGA) is a widely utilized technique in pediatric dentistry and is indicated for a variety of cases such as very young children and children with special healthcare needs (SHCN). In the United Arab Emirates (UAE), there is a paucity of studies relating to this subject.

**Objective:**

To analyze the characteristics of DGA treatment in special healthcare needs and healthy children in the only postgraduate dental hospital in Dubai, UAE.

**Materials and Methods:**

A retrospective analysis was conducted on the electronic records of all Dubai Dental Hospital (DDH) pediatric patients who underwent DGA in the period between January 1^st^, 2016, and 29^th^ of February 2020.

**Results:**

The study population consisted of 98 children. A total of 26 children had a medical condition and were categorized as SHCN. The most common justification for DGA was dental caries and a lack of cooperation due to young age. SHCN patients received significantly more preventive measures and significantly fewer pulp therapies than healthy patients.

**Conclusion:**

We found that the services provided under DGA for healthy pediatric patients differ from those provided to SHCN patients. Frequently missing recall appointments following DGA increased the likelihood of the need for further restorative dental treatment. These results highlight the importance of robust prevention and follow-up programs for children treated under GA.

## 1. Introduction

Dental general anesthesia (DGA) is a widely used technique in pediatric dentistry. It facilitates the delivery of appropriate treatment to children with severe dental caries who cannot cope with treatment in the conventional dental setting due to anxiety or limited cooperation ability [1]. There are several indications for DGA in children but caries is generally the most common cause [2]. Occasionally, some healthy and in special healthcare needs (SHCN) patients require treatment under general anesthesia (GA) due to congenital and medical disorders, dental anomalies (like supernumerary teeth), or dental trauma. SHCN patients suffering from physical, developmental, mental, sensory, behavioral, cognitive, or emotional impairment lack the cooperative skills that are needed to facilitate the delivery of dental treatment. GA provides an alternative option where cooperation from the patient is not required and treatment can be delivered safely [3]. Moreover, patients treated under GA are unconscious and nonresponsive to pain resulting in reduced anxiety towards dental treatments in the future [4]. Several studies have shown that the quality of life has significantly increased for children who went through DGA [5, 6]. Even though some risks are associated with DGA, it remains a safe procedure overall [7]. DGA comes with a high cost and requires unique equipment and a hospital setting; nonetheless, both dentists and parents find it an acceptable way of treating children [8]. Several researchers have examined data from DGA cases and tried to pinpoint the primary causes leading to them [9–11]. Furthermore, factors affecting the success rate of DGA are widely investigated in the literature [12]. One study suggests that treatment planning for complete oral rehabilitation (COR) under GA differed from conventional methods [8].

Therefore, this study aimed to analyze the characteristics of children who underwent DGA for comprehensive oral rehabilitation (COR) in the UAE and to investigate treatment methods delivered and evaluate their outcomes. The null hypothesis was that the medical conditions of the patients will not affect treatment decisions and outcomes when treating children with SHCN.

## 2. Materials and Methods

### 2.1. Study Design and Population

This study was a retrospective descriptive-analytical design. The study protocol was approved by the Mohammad Bin Rashid University Institutional Review Board (Ref: MBRU-IRB-2020-007). Inclusion criteria involved all children who went through COR under GA or any dental treatment that required GA in the period between January 1^st^, 2016, and February 29^th^, 2020, at Dubai Dental Hospital (DDH), UAE. DDH is the only dental hospital in the Emirates of Dubai, UAE, and is the clinical partner of the Hamdan Bin Mohammad College of Dental Medicine, a postgraduate college at Mohammed Bin Rashid University of Medicine and Health Sciences (MBRU). Exclusion criteria included children treated under local analgesia and sedation or those who had GA before or after January 1^st^, 2016, to February 29^th^, 2020.

### 2.2. Data Collection

To acquire the data needed for this analysis, a retrospective review of the electronic records was performed by accessing the digital clinical notes in DDH Dental4Windows™ (D4W) system. Permission was sought by DDH to access these files and anonymous demographic data, such as age, gender, and nationality, were obtained from electronic patient files without any personal identifiers besides the file number. Pre-GA information such as diagnosis, DGA indication, DGA justification, patients' medical history, and the date of the DGA was gathered through the standardized surgical booking form completed by the operator (pediatric dentistry postgraduate student supervised by a consultant pediatric dentist) prior to the operation. Post-GA information such as follow-up attendance rate, postoperative complication, and the need for further treatment was collected from the D4W clinical notes for the subsequent follow-up visits. Details of the surgery were also retrieved using the postoperative discharge form which included the total duration, characteristics of treatments provided (crowns, extractions, and restorations), and adverse events if any.

The data were collected by the principal investigator (MA). Two cycles for intraexaminer reproducibility testing were performed. A random number generator program was used to randomly select 10 patients' records to conduct the kappa statistical analysis of categorical variables. The two cycles were separated by a washout period of 10 days. Intraexaminer reproducibility Kappa coefficient of 0.87 was obtained, which was considered outstanding.

### 2.3. Statistical Analysis

The IBM SPSS software version 25 (SPSS Inc., Chicago, IL, USA) was used to analyze the data using descriptive statistics. The normality of the available data was tested using the Kolmogorov–Smirnov test. Since the data were not normally distributed, the Mann–Whitney *U* test was used as a nonparametric statistical test to compare independent groups at a level of significance set as 5%. While, categorical variables such as age, gender, and medical condition were plotted using frequency tables. Cross tabulations: the chi-square test was used to compare differences between SHCN patients and their healthy peers.

## 3. Results

### 3.1. Study Population

**Figure 1:**
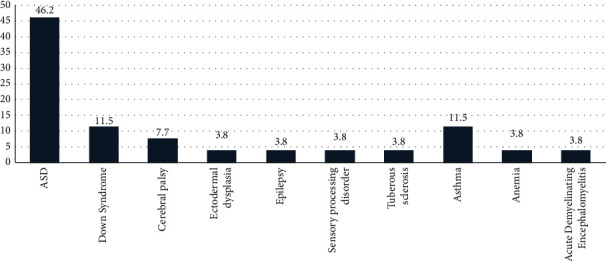
Types of medical conditions in the study population.

The study population consisted of 98 children. More than half of the sample populations were males (*n* = 61, 62.2%) and 37 patients were females (37.8%). Patients' ages ranged 1–15 years with a mean age distribution of 5.4 years and a standard deviation (SD) of ±2.8 years. The age of SHCN patients was significantly higher (7.99 years ± 3.7) than that of healthy patients at the time of the GA (*P* ≤ 0.0001). Medical disorders and special healthcare needs (physical or intellectual) were identified in 26 (26.5%) of the subjects. The frequency of these conditions is summarized in Figure 1. Autism spectrum disorder (ASD) was the most frequently encountered condition with a percentage of 46.2%. Asthma and Down's syndrome were equally identified as the next most common conditions at 11.5%, followed by cerebral palsy (7.7%). Remaining disorders shared the same percentage of 3.8%.

### 3.2. Reasons for DGA

**Figure 2:**
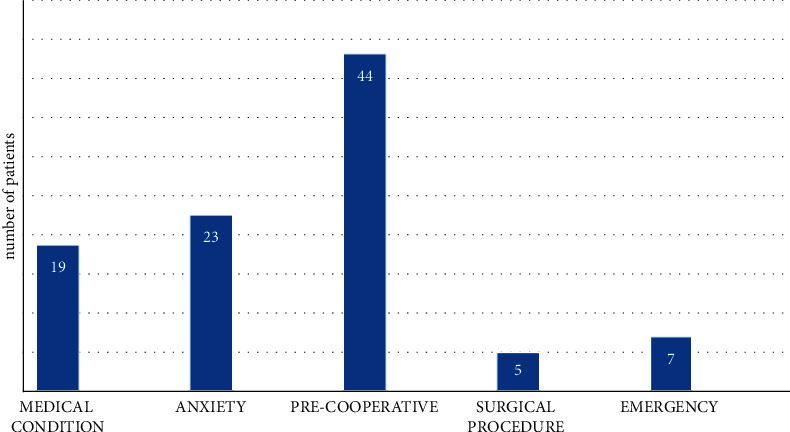
Indications of DGA as recorded in the clinical notes.

Several reasons justified managing these children under GA. Five main categories recorded in the patient's files and included in this study are shown in Figure 2. These included young pre-cooperative children (*n* = 44, 44.9%), with severe anxiety and refusal of standard in-chair treatment (*n* = 23, 23.5%), conditions impeding conventional dental treatment (*n* = 19, 19.4%), emergency (sepsis/pain) requiring immediate intervention (*n* = 7, 7.1%), and complex surgical procedures which required absolute cooperation (*n* = 5, 5.1%).

### 3.3. Dental Diagnosis Leading DGA

Data collected to identify the main dental diagnosis leading to DGA are shown in Table 1. The most common diagnosis was severe early childhood caries (S-ECC; *n* = 85, 86.7%) and facial cellulitis (*n* = 5, 5.1%). Three patients (3%) had a supernumerary tooth, two patients (2%) had facial and dental trauma, while two patients (2%) had periodontal disease, and one patient (1%) was diagnosed with amelogenesis imperfecta.

### 3.4. Type of Treatment Procedures

**Figure 3:**
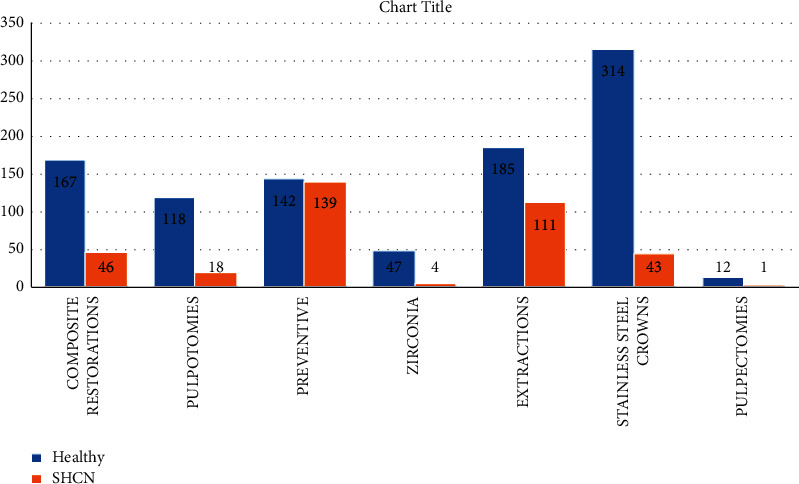
Type of DGA treatment procedures.

As Figure 3 shows, a total of 1347 treatment procedures were performed of which 357 (26.50%) were performed stainless steel crowns (SSCs), 296 (21.97%) were extractions (including surgical), 213 (15.81%) were composite restorations, 136 (10.10%) were primary teeth pulpotomies, 51 (3.79%) were zirconia aesthetic crowns, 13 (0.97%) were primary molar pulpectomies, and the remaining 281 (20.86%) were preventive treatment procedures in the form of sealants, fluoride, and full-mouth prophylaxis. Further analysis of preventive treatment procedures showed that 70.4% of the study sample received full-mouth scaling and polishing at the beginning of the treatment and 51% of the children received topical fluoride varnish during operation. While, impressions for space maintainers were taken for only 14.3% of patients during DGA.

### 3.5. Attendance Rate for Follow-Up Appointments

**Figure 4:**
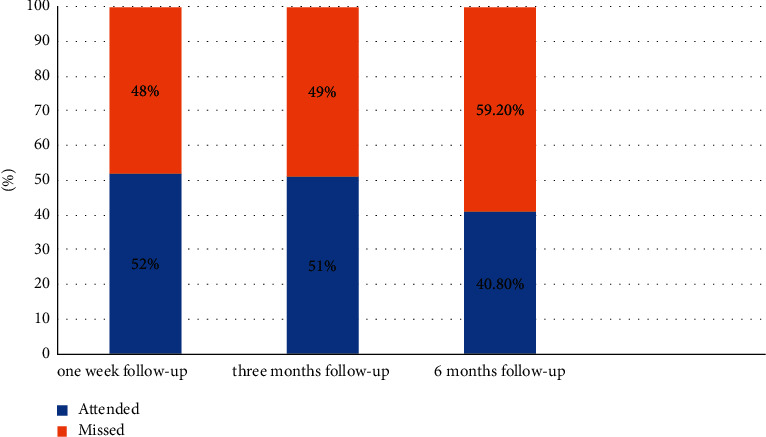
Follow-up attendance rates.

Overall, 27 (28%) patients missed all their follow-up appointments and were never seen after the procedure. The pattern of attendance to the follow-up program was analyzed as shown in Figure 4. Out of the 98 DGAs, almost half 47 (48%) of the patients did not attend their one-week follow-up appointment. Failing to attend the three-month follow-up visit was noticed in almost half of the patients as 48 (49%) missed their follow-up session. Last, upon checking the attendance of the six-month appointment, it was noticed that 58 (59.2%) of patients did not come back for their routine check-ups.

### 3.6. Need for Further Dental Treatment

**Figure 5:**
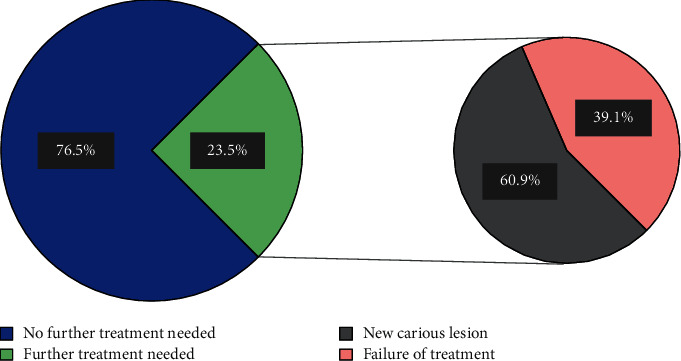
Number of patients requiring further dental treatment following DGA.

Within a 16-month duration following the DGA, 23 (23.5%) patients needed additional dental care, of which 14 (60.9%) were diagnosed with new lesions and 11 (39.1%) were diagnosed with a failure of previously delivered treatment such as recurrent caries, failed fissure sealants, and lost SSCs or restorations (Figure 5).

### 3.7. Correlation between Follow-Up and Further Dental Needs

The records of the 23 patients who received additional dental treatment following the DGA were thoroughly examined to measure their attendance rate to the scheduled follow-up appointments. It was found that 52.2% of these patients missed their one-week visit, while 66.7% missed their three-months appointment and 60.9% did not show to their six-month follow-up. The follow-up attendance rate was correlated to the need for further treatment, and the result was statistically significant at a *P* value of 0.008 using the chi-square test.

### 3.8. Healthy vs. SHCN Patients

As shown in Table 2, the sample consisted of 26 (26.5%) SHCN patients, and 72 (73.5%) were healthy children. The mean age of the two groups at the time of the DGA was found to be significantly different. Healthy patients had a mean age of 4.48 (SD 1.7) years. While, the SHCN group's mean age was 7.9 (SD 3.7) years. When these results were analyzed using the Mann–Whitney test, a significant *P* value of 0.0001 was noted.

Treatment delivered to both groups was investigated and compared. Duration of treatment was not found to be significantly different with a *P* value of 0.143. Extractions, composites, pulpectomies, and zirconia crowns were also not found to be significantly different, with *P* values of 0.170, 0.413, 0.342, and 0.329, respectively. Data analysis showed that SHCN patients received fewer pulpotomies than their healthy peers. This was found to be statistically significant with a *P* value of 0.008. Additionally, receiving SSCs was statistically significantly different (*P*=0.0001), as the healthy patients' group received more SSCs. Last, the data showed that fissure sealants application differed significantly between the two groups. SHCN patients received an average of 3.65 sealants per patient compared to a 1.51 sealants per healthy child. The difference was statistically significant with a *P* ≤ 0.001.

## 4. Discussion

The present study was designed to investigate the characteristics of comprehensive dental treatment provided under general anesthesia in a postgraduate dental hospital in Dubai, UAE. Several studies were conducted worldwide investigating different elements of pediatric DGA [9, 10, 12]. However, to the best of our knowledge, no such study assessing the DGA characteristics had been carried out in the UAE to date.

The sample's mean age was 5.4 years (SD ± 2.8). This value is consistent with the findings of other studies, in which similar results were found [8, 13]. In their study, Heidari et al. concluded that young age and lack of cooperation were the most common reasons for performing dental treatment under GA in children [11]. However, the age of SHCN patients was significantly higher (7.99 years ± 3.7) than that of healthy patients at the time of the GA (*P* ≤ 0.0001). This may suggest that DGA remains the preferred treatment option for patients with SHCN who cannot cope with the conventional dental setting even at an older age.

Moreover, in this study, S-ECC was found to be the most common cause of DGA (85%). These results seem to be consistent with other studies, which found that most of their DGAs were also caused by S-ECC [14–16]. These findings are rather disappointing and suggested that ECC, a preventable disease, remains a burden upon children, families, and the wider society that needs to be resolved. While the intake of simple sugars drives the carious process, the topical and systemic application of fluoride and other preventive measures can attenuate the process. Water fluoridation, if available, is an effective oral health improvement intervention that does not require behavior change by individuals [17].

Most treatment procedures carried out under DGA were a combination of extractions, restorative, and preventive interventions. Preformed SSCs were the predominant modality of restorative treatment over composite restorations and zirconia crowns. This result could be explained by the fact that SSC's have been strongly supported by data to be the most durable and with the highest success rates amongst all restorative materials [18]. In their study, Jamjoom et al. (2008) showed that extractions were very few for patients going through COR under GA [15]. This differs from the findings of the present study, as extractions were higher in number than all types of pulp therapies combined (pulpotomies, pulpectomies, and indirect pulp cap). This may be the result of more severe unrestorable carious teeth. Another possible explanation for this might be the available evidence showing that an aggressive treatment plan should be adopted for pediatric DGA to ensure a successful outcome and prevent the need for a repeat in the future [10, 19]. It is worth mentioning that these DGA procedures were only of a restorative nature provided by postgraduate pediatric dentistry trainees under pediatric dentistry consultants' supervision in contrast to some exodontia DGA procedures which are commonly practiced by specialist pediatric dentists in the UK [20]. Correspondingly, further research is required to establish the success rate of each treatment individually. One complication of premature extraction of primary teeth at a younger age is the definitive loss of space. Bhujel et al. concluded in their study that premature extraction of primary teeth at a young age significantly increased the need for orthodontic treatment in the future [21]. Similarly, a systematic review by Kaklamanos et al. showed that 1.5 mm of space could be lost after premature extraction of first primary molars [22]. This highlights the importance of prevention and the need to preserve these teeth as much as possible.

A significant percentage of patients failed to show their scheduled recall visits following their DGA. This finding matched those observed in earlier studies showing minimal adherence of patients to their scheduled follow-up appointments [15, 23, 24]. A few treated patients were referred for COR under GA by their general practitioner who does not provide the service of GA. It is possible that these patients continued their follow-up sessions with the referring dentist afterward. Owing to this fact, this study was unable to measure the success/failure rate of treatments delivered during DGAs. Patients who attended regularly received preventive interventions, oral hygiene instructions, and dietary advice. Hence, very few required further restorative treatment. However, 23 patients received additional dental treatment following their DGA. A combination of glass ionomer cement (GIC) and SSCs using the Hall technique (HT) was utilized for the delivery of these treatments. A study by Al Halabi et al. showed that this approach could be used as an acceptable alternative to GA for children who are pre-cooperative or uncooperative [25]. Additional options are available for in-chair treatment such as the newly developed bioactive materials. In their study, Lardani et al. concluded that Activa™ which is a bioactive restorative material compromised of both composite and glass ionomers performed similar to composites in a one-year follow-up [26], thus making the material ideal for restoring primary molars when cooperation is limited. Furthermore, a systematic review by Cianetti et al. showed that the use of oscillating and ultrasonic tips in caries removal was less painful and was associated with a lower level of discomfort when compared to the rotary drill [27].

An interesting correlation was found between attendance rate to recall visits and the need for further treatment. It was found that those who needed additional dental treatment missed most of their routine check-up appointments (or were not brought in, WNB). One possible explanation for this finding is that patients who missed their follow-up appointments and WNB did not receive the routinely needed preventive interventions. These results agree with the findings of other studies in which regular follow-up was mentioned as one of the significant factors in the success rates of DGA [10, 28]. This finding may suggest that establishing a rigorous preoperative prevention plan may help prevent the need for further dental treatment following DGA. However, a note of caution is due here, since this study was not designed to assess the factors affecting the need for further dental treatments following DGA. Further research should be undertaken to investigate this topic.

Most of the dental procedures provided during the DGA were extractions and SSCs for both primary and permanent teeth. However, further analysis of treatment modalities between SHCN and healthy children indicated significant differences. The SHCN group seemed to have far fewer pulp therapies than healthy children. This could indicate that the severity of the decay was less in healthy children allowing them performing more pulpal procedures compared to SHCN children where the caries activity was more advanced rendering the success of pulp therapy questionable. Consequently, the operators favored the option of extraction. This phenomenon is not uncommon, where previous studies showed that the child's health status seemed to affect the decision of treatment [29, 30]. Operators tend to provide more radical dental treatments for SHCN patients under DGA to ensure a higher success rate and prevent future complications [31]. Even though the results of this study did not show a significant difference, extractions in the SHCN patients were higher in number than in the healthy group. Moreover, SHCN patients also received significantly fewer SSCs than their healthy peers. This can further support the hypothesis that when caries activity is at an advanced stage, extraction is the favored approach in SHCN children. However, with a small sample size, caution must be applied, as the findings might not apply to the general population. More specifically designed studies with emphasis on decayed, missing, and filled surfaces (DMFS) investigation are required to gain more insight on this topic. On the other hand, the SHCN group received significantly more fissure sealants for both primary and permanent teeth than the healthy patients. This observed increase in the preventive measures amongst SHCN patients could be attributed to the fact that this group of children is at a higher risk of caries development [32]. Furthermore, infections arising from a dental disease may interfere with the control of their medical condition [33]. One more possible explanation is that dental treatment for children with SHCN may require facilities with special equipment to allow the treatment to be delivered safely [34]. Therefore, reducing the morbidity of oral conditions and preventing the development of dental decay following DGA was a major concern prior to commencing the treatment. Additionally, SHCN children DGA was performed at an older age with a higher number of permanent molars that needed sealants. On numerous occasions, the SHCN children are treated under GA to perform proper professional scaling and prophylaxis when they present with heavy calculus deposits. The usual practice for these children is to seal any noncarious teeth under GA.

### 4.1. Limitations of the Study

The following important limitations need to be considered. The principal limitation of this study was the nature of its retrospective design. Thus, the results are based on the accuracy of the recorded information. In addition, this study was conducted in the only postgraduate dental hospital in Dubai. While this represents an important academic aspect of pediatric dentistry, it is not fully representative of Dubai. This is because DGA is carried out in numerous private pediatric dental facilities spread across the city and governmental health centers that provide DGA procedures to children. Therefore, further studies using a different experimental setup should be conducted to better understand this topic.

In summary, this study has expanded our knowledge of the pattern of dental treatment performed for the pediatric population under GA as well as the important factors that might affect the success rate of pediatric DGA. These observed findings are particularly relevant and could be used to help the future planning of COR under GA for children.

The results of this study highlighted the importance of oral health education for parents/guardians of children, especially SHCN children. Moreover, parents' education is vital to establish regular supervision of oral hygiene habits as many children with SHCN might face difficulties maintaining optimum oral care. It is also essential to educate parents/caregivers on the importance of regular attendance to follow-up appointments following the DGA. Moreover, a preoperative prevention protocol consisting of multiple sessions to evaluate oral hygiene improvement could be adopted for children scheduled for COR under GA.

## 5. Conclusions

In the sample of pediatric dental patients treated under dental general anesthesia in a postgraduate dental hospital in Dubai and within the limitation of this study, the following can be concluded:

- Preformed SSCs were the predominant modality of restorative treatment over composite restorations and zirconia crowns
- S-ECC and pre-cooperative stage were the main reasons leading to DGA
- Comprehensive treatment plans, which consisted mainly of dental extractions and fewer pulp therapies, were found to have been conducted in those SHCN children, accompanied by a notable increase in preventive interventions when compared to healthy patients
- A major finding was that many children were not brought-in following DGA and had frequently missed recall appointments. This increased the likelihood of developing new carious lesions and consequently increased the need for further dental treatment.

## Figures and Tables

**Table 1:** Dental diagnosis and the main reason of DGA.

Diagnosis	*N* (%)
S-ECC	85 (86.7)
Cellulitis	5 (5.1)
Supernumerary tooth	3 (3)
Trauma	2 (2)
Periodontal disease	2 (2)
Amelogenesis imperfecta	1 (1)
Total	**98 (100)**

**Table 2:** Age, duration, and type of dental procedure in healthy vs. SHCN patients.

		Age in years	Duration in minutes	Extraction	Composite	Pulpotomy	Pulpectomy	SSC	Zirconia	Sealants
Healthy	*N*	72	72	72	72	72	72	72	72	71
	Mean	4.48	91.45	2.57	2.32	1.64	0.17	4.36	0.65	0.77
	Std. deviation	1.77	26.41	2.37	2.33	1.73	0.63	2.60	1.53	1.51

SHCN	*N*	26	26	26	26	26	26	26	26	26
	Mean	7.99	87.58	4.27	1.77	0.69	0.04	1.65	0.15	3.65
	Std. deviation	3.66	21.39	4.39	1.73	1.01	0.19	1.88	0.46	4.65

Total	*N*	98	98	98	98	98	98	98	98	97
	Mean	5.42	97.14	3.02	2.17	1.39	0.13	3.64	0.52	1.55
	Std. deviation	2.86	26.61	3.11	2.19	1.62	0.55	2.70	1.35	2.99
*P* value	Sig. (2-tailed)	**0.0001**	0.143	0.170	0.413	**0.008**	0.34	**0.0001**	0.329	**≤0.001**

## Data Availability

The data used to support the findings of this study are available from the corresponding author upon request.
